# The comparison of catheter ablation on hard outcomes versus medical treatment for atrial fibrillation patients: A meta-analysis of randomized, controlled trials with trial sequential analysis

**DOI:** 10.1371/journal.pone.0262702

**Published:** 2022-01-19

**Authors:** Jikai Song, Qinggang Zhang, Lifang Ye, Yaru Zheng, Lihong Wang

**Affiliations:** Zhejiang Provincial People’s Hospital, Qingdao University, Hangzhou, Zhejiang, China; The University of Mississippi Medical Center, UNITED STATES

## Abstract

**Background:**

The prevailing view is that ablation does not reduce the incidence of stroke and deaths in atrial fibrillation (AF), and guidelines suggest that long-term anticoagulation is required after ablation, regardless of the success of the procedure. We performed a meta-analysis of recent randomized, controlled trials (RCTs) to verify whether ablation compared with drugs reduced the incidence of stroke and deaths.

**Methods:**

We systematically searched the PubMed, Embase, and Cochrane Central Register of Controlled Trials databases for RCTs of AF catheter ablation (CA) compared to medical therapy (MT). The risk ratio (RR) and weighted mean difference (WMD) with 95% CIs were calculated using a random-effects model. A trial sequential analysis (TSA) was used to further validate the reliability of the primary outcomes.

**Results:**

Seventeen RCTs were included, comprising 5,258 patients (CA, n = 2760; MT, n = 2498). Compared with medical therapy, CA was associated with a reduction in stroke/transient ischaemic attacks (TIAs) (p = 0.035; RR = 0.61 [95% CI, 0.386 to 0.965]; I^2^ = 0.0%) and deaths (p = 0.004; RR = 0.7 [95% CI, 0.55 to 0.89]; I^2^ = 0.0%). CA was associated with improvement in left ventricular ejection fraction (LVEF) (p = 0.000; WMD = 5.39 [95% CI, 2.45 to 8.32]; I^2^ = 84.4%) and the rate of maintenance of sinus rhythm (SR) (p = 0.000; RR = 3.55 [95% CI, 2.34 to 5.40]; I^2^ = 76.7%).

**Conclusions:**

CA for AF had more favourable outcomes in terms of stroke/TIAs, deaths, change in LVEF, and the maintenance of SR at the end of follow-up compared to MT. Besides, the TSA results supported this conclusion.

## Introduction

Atrial fibrillation (AF), the most common persistent arrhythmia in clinical practice, has been called the "cardiovascular epidemic of the 21st century" and is increasing in prevalence worldwide [1]. Stroke events due to AF cause a heavy financial and emotional burden on patients, as well as on society [2]. In particular, strokes carries prognostic implications in terms of mortality and ongoing morbidity [3].

Currently, ablation is a very effective treatment for atrial fibrillation; however, the prevailing view is that ablation does not reduce the incidence of stroke and deaths. Hence, the current guidelines indicate that long-term anticoagulation is required after ablation, regardless of the success of the procedure. Current guidelines support that, following cardioversion for AF of any duration, the decision about long-term anticoagulation therapy should be based on the thromboembolic risk profile (class IC recommendation) [4]. Why is this the case? It is mainly because of the lack of randomized, controlled studies (RCTs) in this area.

Our group performed the same meta-analysis 5 years ago [5], but compared to medical treatment, the meta-results on AF ablation at that time did not reduce stroke and deaths. However, with the accumulation of experience and the maturity of the technique, the success rate of ablation has been increasing, and the incidence of stroke and deaths after ablation has been gradually decreasing. At the same time, there has been an increasing number of recent RCTs on stroke incidence after ablation over the last 5 years, so we decided to perform further meta-analysis by combining recent RCTs. If we can indeed find a reduction in stroke and deaths incidence, it will not only change the guidelines but will also provide a significant benefit to patients with AF. Our latest meta-results differ from our previous meta-results in that they show a statistically significant reduction in stroke and deaths incidence, supporting our latest view and suggesting the outcome that recent innovations in atrial fibrillation ablation techniques have indeed affected the incidence of stroke and deaths in atrial fibrillation.

## Methods

The Preferred Reporting Items for Systematic Reviews and Meta-analyses (PRISMA) guidelines were strictly followed in this meta-analysis [6], which was registered on PROSPERO on December 23, 2020, under the number CRD42020220610. We last updated our search on February 7, 2021, to the extent that no new trials met the inclusion criteria for our meta-analysis. We have updated PROSPERO to accurately reflect our search updates.

### Search strategy

The PubMed, Embase, and Cochrane Central Register of Controlled Trials databases were searched by two independent investigators (SJK and ZQG) without language restriction from January 1, 2010, to December 31, 2020. The goal of the search was to dig deep to find all RCTs comparing ablation therapy to conventional drug therapy in patients with atrial fibrillation. Specialized medical librarians assisted us in developing our search strategy, which included four key search terms: atrial fibrillation, catheter ablation, medical therapy, and randomized controlled trial (S1–S3 Tables). We referred to the search strategy through the Cochrane Library review to refine our search formula. We checked the reference lists of articles in the referenced articles for literature completeness, as well as other relevant studies. Only studies issued in English with the full text available were included.

### Study selection and outcomes

We included RCTs comparing ablation with pharmacological treatment of atrial fibrillation. Randomized controlled trials were limited to those that report any of the following long-term outcomes: deaths related to ablation or pharmacological treatment of atrial fibrillation during follow-up and stroke/transient ischaemic attack (TIA) as a nonsurgical correlation. In addition, to minimize the risk of publication bias, as well as the potential degree of heterogeneity, we strictly controlled the inclusion of RCTs≥50 patients and a minimum of six months of follow-up. For those outcomes with multiple time points for reporting, we used the longest follow-up time point. After careful consideration, we excluded the following articles: case reports, observational studies, reviews, expert opinions, and studies without randomized groupings.

The primary outcomes of interest were deaths and stroke/TIA. The secondary outcome was the change in LVEF and the maintenance of SR at the end of follow-up.

### Data extraction and quality evaluation

Two investigators (SJK and ZQG) performed the data extraction independently and designed and used a predeveloped data extraction form. From the included studies, we gathered the following data: crossover, study period, ablation strategy, follow-up duration, baseline study characteristics, risk factors, procedure specifics, and outcome measures.

To assess the included studies, we used the widely used Cochrane Collaboration tool for assessing the risk of bias [7]. Notably, the two researchers independently evaluated the selected studies on seven indicators: random sequence generation, allocation concealment, blinding of participants and personnel, blinding of outcome assessment, incomplete outcome data, selective reporting, and other bias. All of the evaluation indicators with different opinions were resolved through a rigorous discussion.

### Statistical analysis

A part of the meta-analysis was conducted using the inverse variance (IV) random-effects model in STATA software, version 14.0 (Stata Corporation, College Station, TX, USA), to quantify Egger’s test of publication bias and the synthesis of primary and secondary outcome indicators. For the other part of the meta-analysis, we used the DerSimonian-Laird (DL) random-effects model [8] in STATA software, version 16.0 (STATA Corporation, College Station, TX, USA), to qualitatively analyse the contour-enhanced funnel plot for publication bias and to perform sensitivity analyses of the synthetic results for the primary and secondary outcome indicators.

We used the risk ratio (RR) for dichotomous outcomes, while for continuous outcomes, we used the weighted mean difference (WMD) with 95% confidence intervals (CIs) in both cases. Considering a weight of zero, forest plots did not indicate studies of zero occurrences in either the intervention group or the control group. For the meta-analysis of continuous variables, we converted the median and interquartile spacing into the form of the mean and standard deviation from reliable formulas [9, 10]. We assessed the heterogeneity among the overall studies in the forest plot by the following two metrics: the chi-square test for homogeneity (a relatively large heterogeneity is indicated if p<0.1) and the I^2^ statistic (a result of 50% or greater would suggest potentially large heterogeneity) [11]. Publication bias was analysed qualitatively by contour-enhanced funnel plots, and Egger’s test was utilized to aid in the quantitative analysis of publication bias. Sensitivity analysis of the outcome indicators was performed by trim-and-fill analysis to determine the stability of the results.

To further confirm the reliability of the primary outcome indicators of stroke/TIA and death, we performed a trial sequential analysis (TSA) [12] using a random-effects (DL) model. We used conventional confidence intervals of 95%, a two-sided boundary type with type 1 error of 5%, and an information axis with sample size. For TSA of stroke/TIA, we used a relative risk reduction of 38.49% (low bias-based) and an incidence in the control arm of 2%. For TSA of deaths, we used a relative risk reduction of 30.03% (low bias-based) and an incidence in the control arm of 6.1%. The heterogeneity correction for both TSAs was set to model variance based. Trial Sequential Analysis software, version 0.9.5.10 beta (Copenhagen Trial Unit, Centre for Clinical Intervention Research, Rigshospitalet, Copenhagen, Denmark, https://www.ctu.dk/tsa), was used for this analysis.

## Results

### Characteristics of the studies

**Fig 1:**
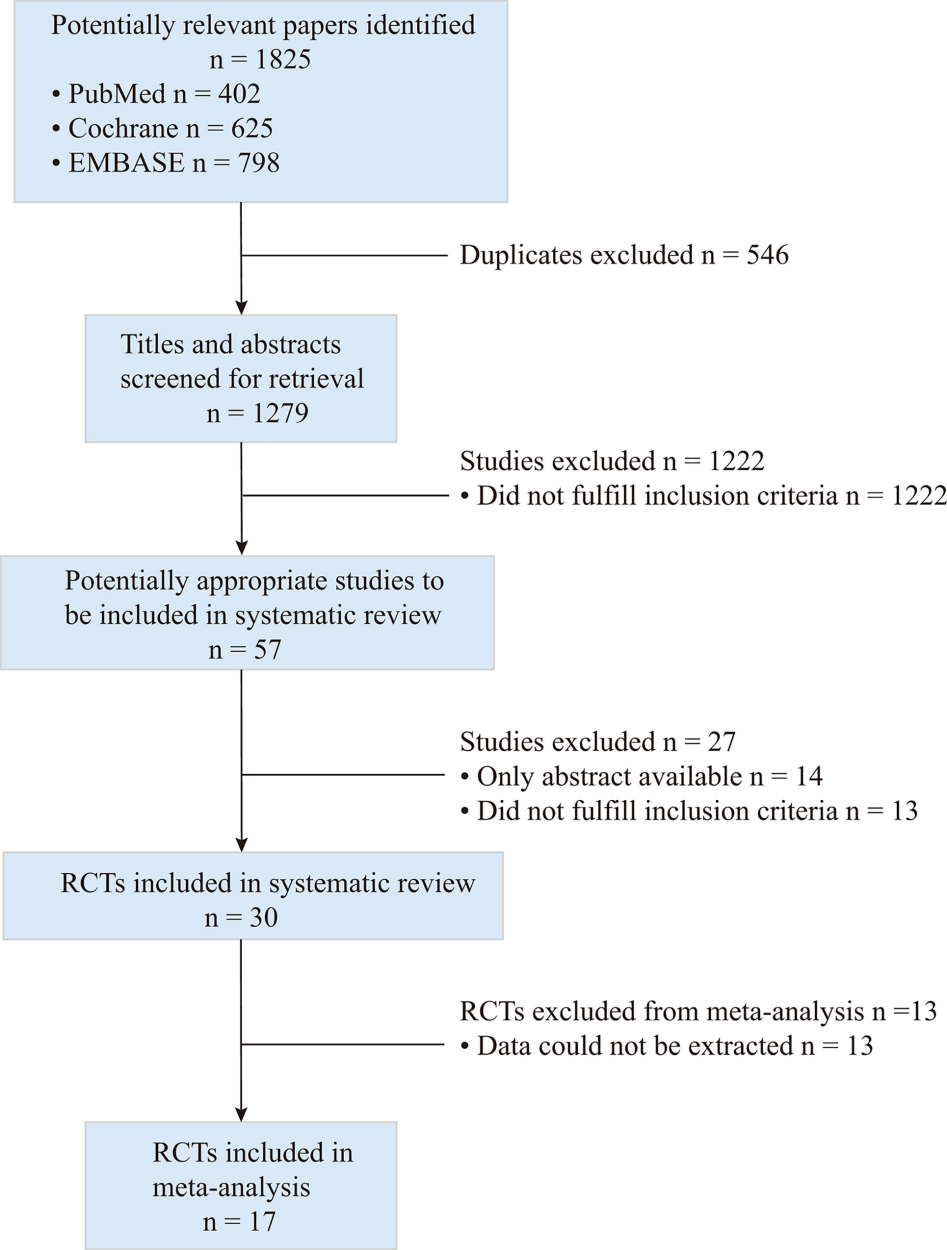
Flow diagram of studies included in the meta-analysis.

Through a scan of the three databases, a total of 1825 possible studies were discovered. Of these studies, a total of 546 documents were excluded due to duplication, and another 1222 documents were omitted because they did not meet the inclusion and exclusion criteria. Following a full-text review of the remaining 30 publications, 17 RCTs with a total of 5258 participants were chosen [13–29]. (Fig 1)

Tables 1 and 2 provide a summary of the study characteristics of the randomized trials included in the meta-analysis, as well as baseline patient information. Overall, the mean age of patients in the trials ranged from 54 to 64 years old, the mean LVEF among the study populations was 50.1%, and the LA diameter was 44.0. Overall, women accounted for approximately 30% of the total number of patients, and the predominant ablation strategy was PVI ± additional ablation. In terms of switching rate, the rate of switching to the ablation group was higher than that of switching to the drug treatment group. The study follow-ups ranged from 6 to 60 months.

**Table 1:** Study characteristics of the randomized trials included in the meta-analysis.

References	Crossover, n (%)		CA group			MT group			AT (months)		Follow-up (months)
	To ablation	To drug therapy	Sample size	Age (year)	Female (%)	Sample size	Age (year)	Female(%)	CA	MT	
MacDonald et al.2010	NR	NR	22	62±6.7	23	19	64±8.3	21	NR	NR	6
Wilber et al.2010	59	4.7	106	56±9	31.1	61	56±13	38	3	NR	9
Pappone et al.2011	42	None	99	55±10	NR	99	57±10	NR	NR	NR	48
Nielsen et al.2012	36	8.9	146	56±9	32	148	54±10	28	NR	NR	24
Packer et al.2013	79	None	163	57±9	23.3	82	56±9	22	3	NR	12
Jones et al.2013	NR	NR	26	64±10	19	26	62±9	8	NR	NR	12
Mont et al.2013	0	35.7	98	55±9	22.5	48	55±9	23	1	NR	12
Hunteret al.2014	NR	NR	26	55±12	3.8	24	60±10	4.2	3	3	12
Hummel et al.2014	60	None	138	60±8	16.7	72	61±8	16.7	6	6	6
Morillo et al.2014	42.6	9.1	66	56±9	22.7	61	54±12	26.2	3	NR	24
Biase et al.2016	NR	NR	102	62±10	25	101	60±11	27	NR	NR	24
Prabhu et al.2017	4.5	None	33	59±11	6	33	62±9.4	12	NR	NR	6
Marrouche et al.2018	9.8	15.6	179	63±11.2	13	184	64±13.1	16	NR	NR	60
Packer et al.2019	27.5	26.5	1108	67±7.4	37.3	1096	67±7.4	37	3	NR	60
Wu et al.2020	None	None	327	64.8±12.6	33.3	321	64.4±13.6	36.8	3	NR	60
Kuck et al.2020	12.2	None	102	67.8±4.8	57.8	108	67.6±4.6	58	NR	NR	36
Jason et al.2020	24.2	5.8	154	57.7±12.3	27.3	149	59.5±10.6	31.5	NR	NR	12

Age is given as mean ± SD; CA, catheter ablation; MT, medical treatment; AT, minimum anticoagulation time after ablation or antiarrhythmic drug therapy; NR; not reported.

**Table 2:** Patient information for the included studies.

References	Patients, n		Ablation strategy	Randomized patients, n		Stroke/TIA, n		Death, n		LVEF % at baseline, mean(SD)		LA diameter mm at baseline, mean(SD)	
	Screened	Enrolled		CA	MT	CA	MT	CA	MT	CA	MT	CA	MT
MacDonald et al.2010	325	41	PVI ± additional ablation	22	19	1	0	NR	NR	16.1 (7.1)	19.6 (5.5)	NR	NR
Wilber et al.2010	5378	167	PVI ± additional ablation	106	61	0	0	1	0	62.3 (2)	62.7 (2)	40 (1.1)	40 (1.5)
Pappone et al.2011	334	198	PVI ± additional ablation	99	99	0	0	0	0	60 (8)	61 (6)	40 (6)	38 (6)
Nielsen et al.2012	294	294	PVI ± additional ablation	146	148	1	1	2	4	NR	NR	40 (6)	40 (5)
Packer et al.2013	304	245	PVI ± additional ablation	163	82	7	0	1	0	60 (6)	61 (6)	40 (5)	41 (6)
Jones et al.2013	101	52	PVI ± additional ablation	26	26	0	1	1	0	22 (8)	25 (7)	50 (6)	46 (7)
Mont et al.2013	152	146	PVI ± additional ablation	98	48	0	0	0	0	61.1 (8.8)	60.8 (9.7)	41 (4.6)	42.7 (5.1)
Hunter et al.2014	390	50	PVI ± additional ablation	26	24	1	1	0	1	31.8 (7.7)	33.7 (12.1)	52 (11)	50 (10)
Hummel et al.2014	242	210	PVI ± additional ablation	138	72	1	0	0	0	54.7 (7.1)	54.9 (6.7)	45 (5)	46 (5)
Morillo et al.2014	127	127	PVI ± additional ablation	66	61	0	0	0	0	61.4 (4.8)	60.8 (7)	40 (5)	43 (5)
Biase et al.2016	203	203	PVI ± additional ablation	102	101	NR	NR	8	18	29 (5)	30 (8)	47 (4.2)	48 (4.9)
Prabhu et al.2017	301	66	PVI ± additional ablation	33	33	NR	NR	NR	NR	32 (9)	34 (8)	48 (5.5)	47 (8.2)
Marrouche et al.2018	3013	398	PVI ± additional ablation	179	184	5	11	24	46	31.8 (9.7)	31.9 (7.5)	49 (6.7)	51.3 (4.5)
Packer et al.2019	2204	2204	PVI ± additional ablation	1108	1096	3	7	58	67	NR	NR	NR	NR
Wu et al.2020	1024	652	PVI ± additional ablation	327	321	14	23	5	5	53.3 (9.3)	51.9 (9.4)	45 (8.5)	46 (7.8)
Kuck et al.2020	1237	255	PVI ± additional ablation	102	108	0	1	NR	NR	61.8 (5.8)	62.3 (5.2)	42 (6.1)	43.4 (5.6)
Jason et al.2020	303	303	PVI ± additional ablation	154	149	0	1	NR	NR	59.6 (7)	59.8 (7.6)	39.5 (5)	38.1 (6.5)

CA, catheter ablation; MT, medical treatment; NR, not reported; LVEF, left ventricular ejection fraction; LA, left atrium; TIA, transient ischemic attacks.

### Drugs and technology

In terms of medical treatment, the drugs mainly included class I and class III antiarrhythmic drugs. If atrial fibrillation treatment failed, the next step was adjuvant ablation therapy. In the atrial fibrillation ablation group, there were two trial groups that were not treated with antiarrhythmic drugs prior to enrolment [16, 22]. Patients with recurrence of atrial fibrillation after ablation were treated with a second ablation. The ablation technique was pulmonary vein ablation combined with ablation of linear lesions in the left and right atria, ostia of the pulmonary veins, and cavotricuspid isthmus. The use of additional lesion ablations outside the pulmonary vein region was left to the discretion of the surgeon. Oral anticoagulation had to be given orally for at least 3 weeks prior to atrial fibrillation ablation and for at least 3 months after the procedure, with subsequent anticoagulation to be determined on a patient-by-patient basis.

### Follow-up and withdrawals

By the end of the study, 635 of a total of 2,632 people in the medication group had switched to the CA process after failing on medication or failing to tolerate antiarrhythmic drugs (AADs). The duration of the follow-ups ranged from 6 to 60 months in all studies. Sixty-five patients were lost to follow-up, 8 patients were excluded, and 224 patients withdrew.

### End points

**Fig 2:**
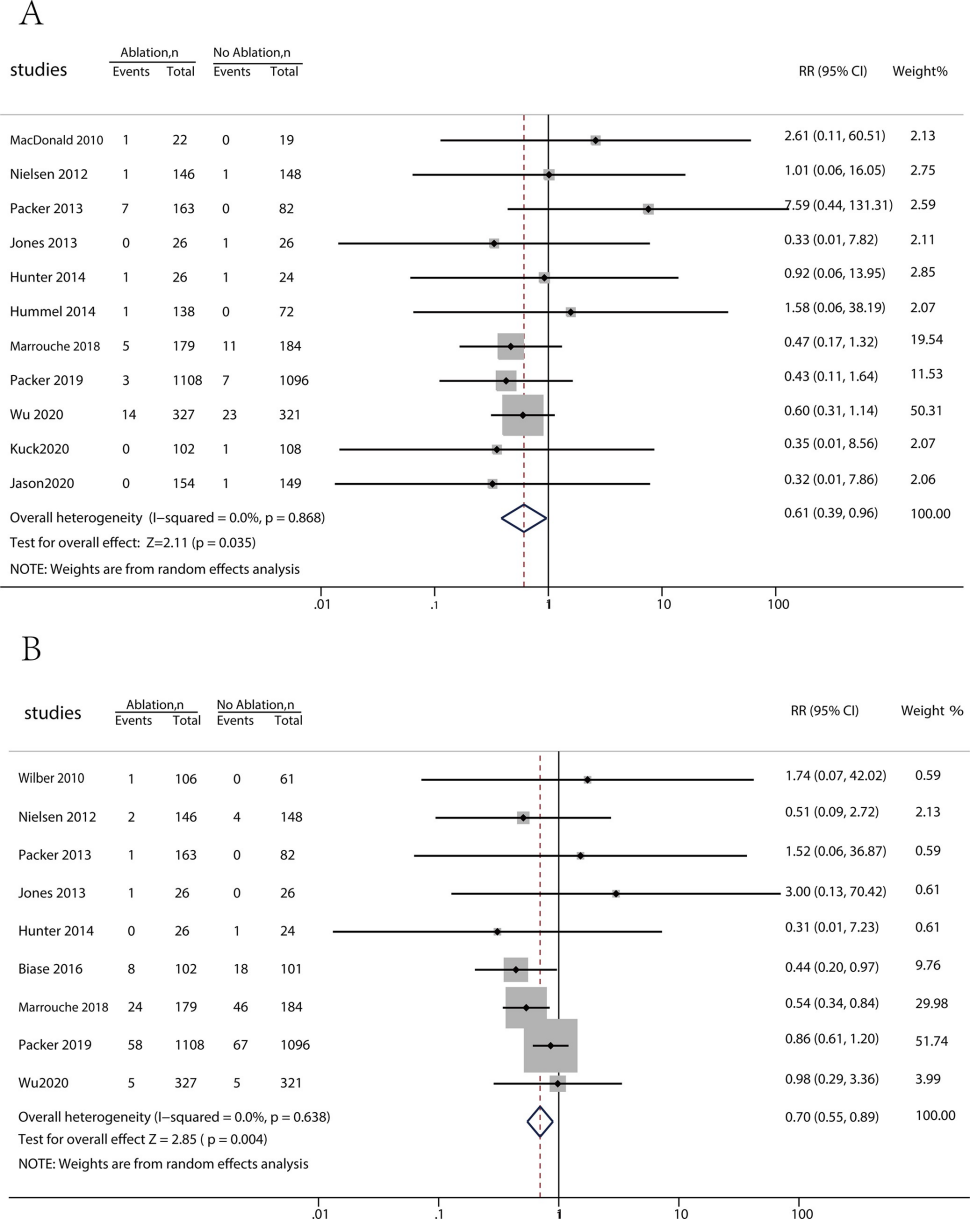
(A) Forest plot displaying risk ratio (RR) for stroke/TIA (B) Forest plot displaying risk ratio (RR) for deaths.

**Fig 3:**
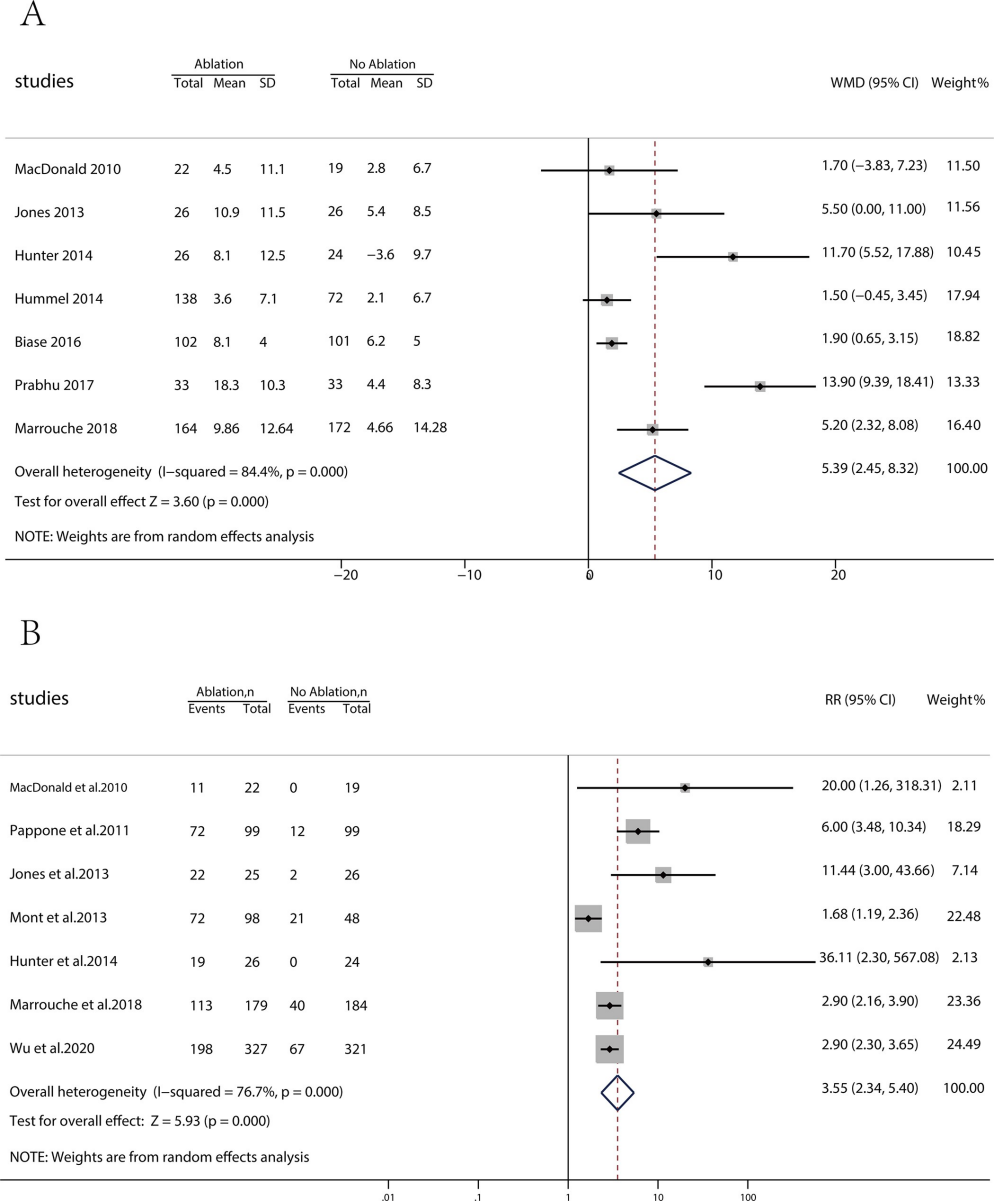
(A) Forest plot displaying weighted mean difference (WMD) for the change in LVEF. (B) Forest plot displaying risk ratio (RR) for the maintenance of sinus rhythm (SR) at the end of follow-up.

Regarding the indicator of stroke/TIA, the meta-analysis findings of atrial fibrillation ablation therapy versus drug therapy synthesis were significantly different (p = 0.035; RR = 0.61 [95% CI, 0.386 to 0.965]; I^2^ = 0.0%) (Fig 2A). The meta-analysis also showed significant superiority in the indicator of death (p = 0.004; RR = 0.7 [95% CI, 0.55 to 0.89]; I^2^ = 0.0%) (Fig 2B), the change in LVEF (p = 0.000; WMD = 5.39 [95% CI, 2.45 to 8.32]; I^2^ = 84.4%) (Fig 3A), and the maintenance of SR at the end of follow-up (p = 0.000; RR = 3.55 [95% CI, 2.34 to 5.40]; I^2^ = 76.7%) (Fig 3B).

Sensitivity analysis (trim-and-fill analysis using the random-effects method in a nonparametric setting) of the outcome indicators showed stable results (Table 3); statistically significant log RR (Log RR for stroke/TIA = -0.602 [95%CI, -1.035 to -0.170]; Log RR for deaths = -0.371 [95%CI, -0.614 to -0.129]; Log RR for sinus rhythm = 1.183 [95%CI, 0.759 to 1.608]) and WMD (WMD for the change in LVEF = 5.39 [95%CI, 2.45 to 8.35]) were still obtained for all of the studies after attribution to trim-and-fill. There was no heterogeneity (I^2^ = 0.0%) in the primary outcome indicators. Although significant heterogeneity (I^2^ = 84.4%, I^2^ = 76.7%) was observed for the secondary outcome indicator (change in LVEF, the maintenance of SR at the end of follow-up), favourable results in LVEF and sinus rhythm with catheter ablation were observed across all of the trials. The majority of the articles in this meta-analysis were of high quality, as measured by the Cochrane Collaboration’s tool, indicating that the study had a low risk of bias (Table 4).

**Table 3:** Sensitivity analyses (nonparametric trim-and-fill analysis).

Outcomes	Number of Studies			Log RR		WMD		95% CI	
	Observed	Imputed	Observed + Imputed	Observed	Observed + Imputed	Observed	Observed + Imputed	Observed	Observed + Imputed
Deaths	13	2	15	-0.357	-0.371			(-0.601, -0.113)	(-0.614, -0.129)
Stroke/TIA	15	3	18	-0.486	-0.602			(-0.932, -0.039)	(-1.035, -0.170)
Sinus rhythm	7	2	9	1.268	1.183			(0.849, 1.686)	(0.759, 1.608)
Change in LVEF	7	0	7			5.39	5.39	(2.45, 8.35)	(2.45, 8.35)

RR, risk ratio; WMD, weighted mean difference.

**Table 4:** Risk of bias assessment.

Study	Random sequence generation	Allocation concealment	Blinding of participants and personnel	Blinding of outcome assessment	Incomplete outcome data	Selective reporting	Other bias
MacDonald et al.2010	Low	Low	Unclear	Low	Low	Low	Unclear
Wilber et al.2010	Low	Unclear	Unclear	Unclear	Low	Low	Unclear
Pappone et al.2011	Unclear	Unclear	Low	Low	Low	Low	Unclear
Nielsen et al.2012	Low	Low	Low	Low	Low	Low	Unclear
Packer et al.2013	Unclear	Unclear	Unclear	Low	Low	Low	Unclear
Jones et al.2013	Low	Unclear	Low	Low	Low	Low	Unclear
Mont et al.2013	Unclear	Unclear	Low	Low	Low	Low	Unclear
Hunter et al.2014	Low	Low	Low	Low	Low	Low	Unclear
Hummel et al.2014	Unclear	Unclear	Unclear	Low	Low	Low	Unclear
Morillo et al.2014	Unclear	Unclear	Unclear	Low	Low	Low	Unclear
Biase et al.2016	Unclear	Low	Low	Low	Unclear	Low	Unclear
Prabhu et al.2017	Low	Low	Low	Low	Low	Low	Unclear
Marrouche et al.2018	Low	Unclear	Unclear	Low	Low	Low	Unclear
Packer et al.2019	Low	Low	Low	Unclear	Low	Low	Unclear
Wu et al.2020	Low	Unclear	Low	Low	Low	Low	Unclear
Kuck et al.2020	Low	Low	Unclear	Low	Low	Unclear	Unclear
Jason et al.2020	Low	Low	Low	Low	Unclear	Unclear	Unclear

### Publication bias

Qualitatively, the contour-enhanced funnel plot did not see significant publication bias regarding the outcome indicators stroke/TIA and deaths (S1 and S2 Figs). Quantitatively, Egger’s test showed that publication bias remained nonexistent for both stroke/TIA (p = 0.247) and death (p = 0.821) (S3 and S4 Figs). Since there were fewer than 10 papers included, the statistical analysis did not include publication bias testing for the improvement in LVEF and the maintenance of SR at the end of follow-up. Based on the quantitative contour-enhanced funnel plot and qualitative Egger’s test, we have strong evidence that the included studies were very comprehensive and generated statistically credible results.

### TSA findings

**Fig 4:**
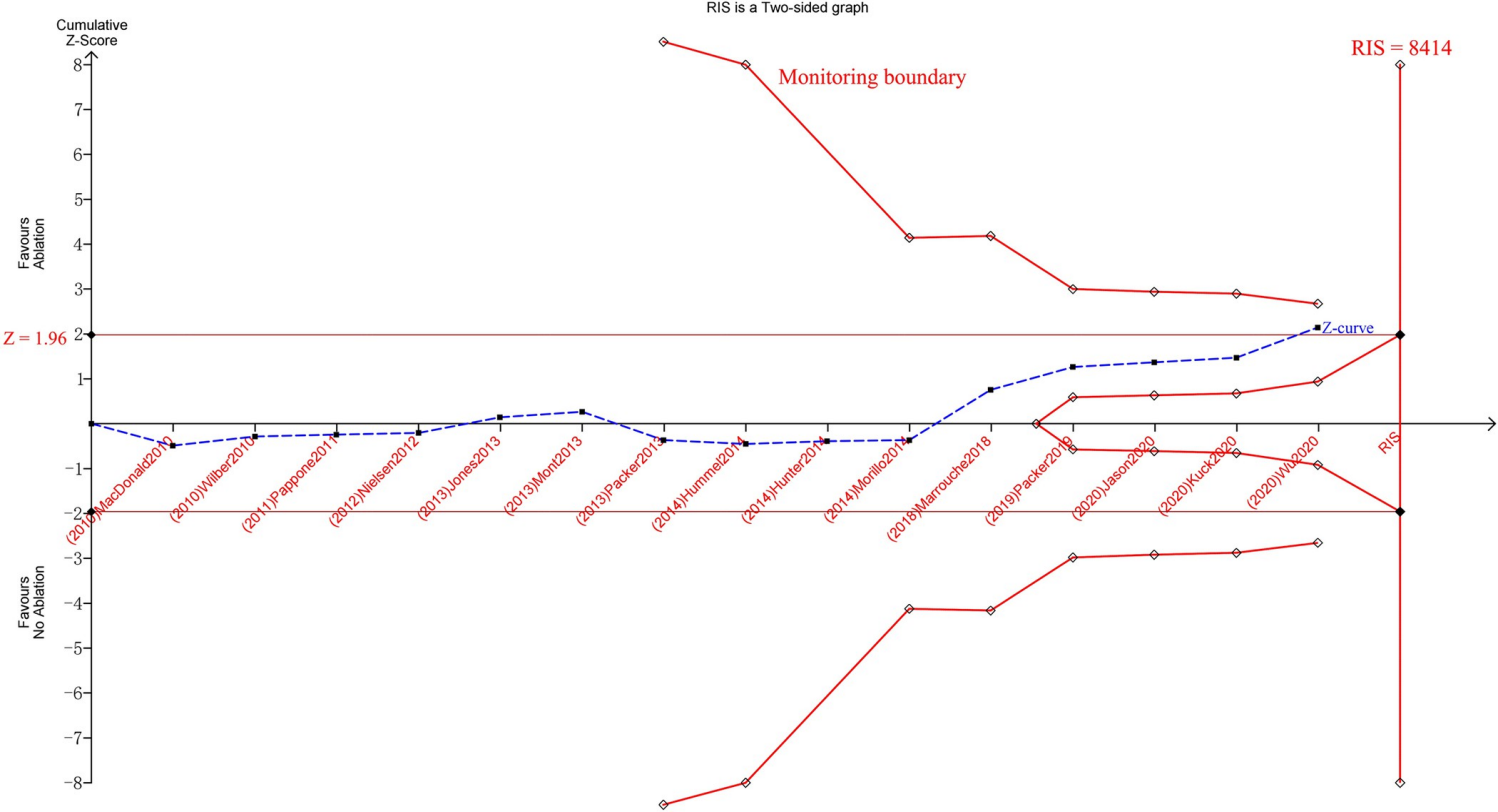
Trial Sequential Analysis (TSA) for stroke/Transient Ischemic Attacks (TIA).

For both primary and secondary outcome indicators, we performed TSA to further validate the reliability of the results [30]. Per the TSA analysis, for three outcome indicators—deaths, the change in LVEF, and the maintenance of SR at the end of follow-up—the respective TSA crossed the traditional Z-curve and monitoring boundary and achieved the required information size (RIS) (S5–S7 Figs), contributing to assessing the strong certainty and precision of these two outcome indicators. For the outcome indicator stroke/TIA, TSA crossed the traditional Z-curve but did not cross the monitoring boundary and did not reach the RIS (Fig 4), contributing to the assessment of moderate certainty and overall moderate precision.

## Discussion

AF ablation treatment significantly reduced the rate of stroke/TIA and deaths and improved LVEF and the maintenance of SR compared to drug treatment. Although previous meta-analyses have found similar results [31–33] regarding deaths and changes in LVEF, this meta-analysis of the positive outcome of stroke/TIA is the first to be reported. This meta-analysis included 17 reliable RCTs from 2010 to 2020 for the analysis, and we performed literature screening and data statistics through a rigorous division of labour. In addition, tests for publication bias, sensitivity analysis of the results, and TSA of the outcome indicators were performed. Layers of data analysis ensured the reliability and stability of our results. Hence, our research is the most comprehensive and up-to-date meta-analysis of the safety and efficacy of ablation for atrial fibrillation treatment.

The indications for CA have gradually extended from paroxysmal AF to persistent AF and long-standing persistent AF. With respect to stroke/TIA, deaths and changes in LVEF, it is crucial to contrast the two rhythm control strategies (catheter ablation vs. medical treatment) with the actual requirements [27]. In this meta-analysis, we found that catheter ablation reduced the risk of stroke/TIA better than drug therapy. A recently published meta-analysis [31] did not yield statistically significant differences in terms of stroke/TIA (RR 0.56, [95% CI 0.23 to 1.36]), primarily because the number of RCTs included regarding this outcome indicator was only four[14, 17, 20, 25]. Correspondingly, this meta-analysis consisted of 17 RCTs. The results of this analysis correlate well with two studies (Wu et al. 2020 [27] and Packer et al. 2019 [26]), accounting for 12.3% and 41.9% of participants in all 17 studies, respectively. This meta-analysis yielded a greater association of positive results with the study of Wu et al., with a weight of 50.31%. Nevertheless, Packer et al. reported in the CABANA study that atrial fibrillation ablation therapy did not result in a significant difference in the outcome indicator stroke/TIA compared to drug therapy. In the CABANA report, 102 (9.2%) of the ablation patients did not have ablation, while 301 (27.5%) of the drug therapy patients had ablation. In Wu et al.’s study, however, all of the participants in the ablation study reported ablative treatment, while those who were medically treated did not switch to the ablation group. These factors might account for the difference in the results of the two studies.

Reduced burden of atrial fibrillation, better-maintained sinus rhythm at the end of follow-up, improved LVEF, and decreased LA diameter are high probability, significant factors contributing to favourable outcomes of catheter ablation. In particular, reduced LVEF is a risk factor for stroke and death. The trial of Marrouche et al. (2018) [25] reported a lower AF burden with catheter ablation (20% and 25% at the 12- and 60-month follow-ups, respectively) than with drug therapy (50% and 65% at the 12- and 60-month follow-ups, respectively). Similarly, Prabhu et al. (2017) [24] reported a mean AF burden at 6 months of 1.6%±5.0% in the AF ablation group, compared with 100% in the drug therapy group. Seven RCTs documented improvement in LVEF, with a mean improvement of 5.39% (Fig 3A). Wu et al. (2020) reported a noticeable reduction in LA diameter in the ablation group compared to the baseline data (1 year: 43 ± 9.1 vs. 45 ± 8.5 mm, P = 0.004; end of study: 42 ± 9.3 vs. 45 ± 8.5 mm, P< 0.001) but not in the medical treatment group (1 year: 46 ± 9.4 vs. 46 ± 7.8 mm, P = 1.00; end of study: 45 ± 9.5 vs. 46 ± 7.8 mm, P = 0.151). The above data echoed the change in LVEF and the reduction in AF burden, suggesting that CA reversed atrial structural remodelling. The combination of these factors could have contributed to the favourable results of this meta-analysis for the primary outcome indicators of stroke/TIA and deaths.

### Limitations

Several limitations merit mention. First, in this study, despite the inclusion of 17 RCTs, the results of our meta-analysis were driven primarily by 4 studies (Di Biase [23], Marrouche [25], Packer [26], Wu [27]). However, the beneficial outcomes of catheter ablation were consistent across most of the trials. Second, in the TSA analysis on stroke/TIA, TSA crossed the traditional Z-curve but did not cross the monitoring boundary and did not reach the required information size, contributing to the assessment of moderate certainty and overall moderate precision. The above scenario requires subsequent RCTs to be added to yield much more reliable results (Required Information Size, 8414). Third, patient selection bias could have been a potential influencing factor since patients who chose to receive ablative therapy might have been healthier than those who received only drug therapy. Notably, the average age of the patients in the trials was 54 to 64 years old, which might be problematic for extrapolating the results to the elderly. Fourth, in all 17 studies, the proportion of female patients was approximately 28%, with an imbalance between men and women. Hence, whether bias is present when extrapolating the results to female patients must also be considered.

## Conclusion

In conclusion, this meta-analysis illustrated that ablation therapy for AF had more favourable outcomes in terms of stroke/TIA, deaths, change in LVEF, and better-maintained sinus rhythm at the end of follow-up compared to medical therapy. In addition, long-term anticoagulation might not be necessary after AF catheter ablation. Considering that the TSA results of stroke/TIA were only of moderate certainty and overall moderate precision, more large-scale clinical research trials to further validate these conclusions are necessary.

## Supporting information

S1 Checklist (DOC)

S1 Fig Contour-enhanced funnel plot for stroke/transient ischaemic attacks (TIAs). (TIF)

S2 Fig Contour-enhanced funnel plot for deaths. (TIF)

S3 Fig Egger’s publication bias plot for stroke/transient ischaemic attacks (TIAs). (TIF)

S4 Fig Egger’s publication bias plot for deaths. (TIF)

S5 Fig Trial sequential analysis (TSA) for deaths. (TIF)

S6 Fig Trial sequential analysis (TSA) for the change in LVEF. (TIF)

S7 Fig Trial sequential analysis (TSA) for the maintenance of sinus rhythm at the end of follow-up. (TIF)

S8 Fig (TIF)

S9 Fig (TIF)

S1 Table PubMed search strategy for trials comparing atrial fibrillation ablation with drug therapy. (DOCX)

S2 Table Cochrane Central Register of Controlled Trials search strategy for trials comparing atrial fibrillation ablation with drug therapy. (DOCX)

S3 Table Embase search strategy for trials comparing atrial fibrillation ablation with drug therapy. (DOCX)

S1 File (XLSX)

## Abbreviations

AF: atrial fibrillation
RCTs: randomized, controlled trials
CA: catheter ablation
MT: medical therapy
RR: risk ratio
TSA: trial sequential analysis
LVEF: left ventricular ejection fraction
SR: sinus rhythm
AADs: antiarrhythmic drugs
WMD: weighted mean difference
CIs: confidence intervals
TIA: transient ischaemic attack
