# Supplementary figures and images for: The comparison of catheter ablation on hard outcomes versus medical treatment for atrial fibrillation patients: A meta-analysis of randomized, controlled trials with trial sequential analysis
Source: PLoS One. 2022 Jan 19;17(1):e0262702. doi: 10.1371/journal.pone.0262702 (PMC8769301; doi:10.1371/journal.pone.0262702)

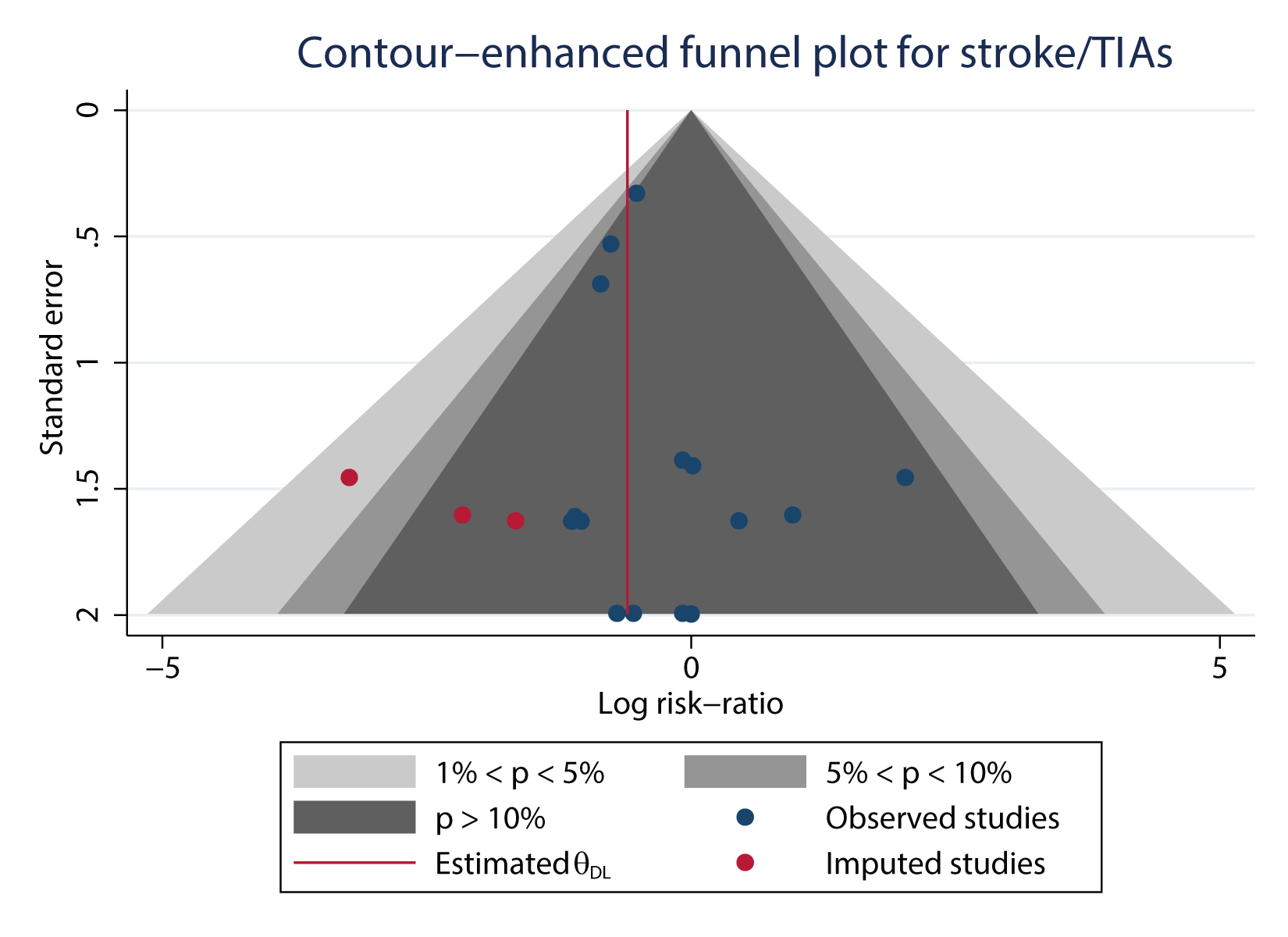

Supplement: S1 Fig — (TIF) [file pone.0262702.s002.tif]

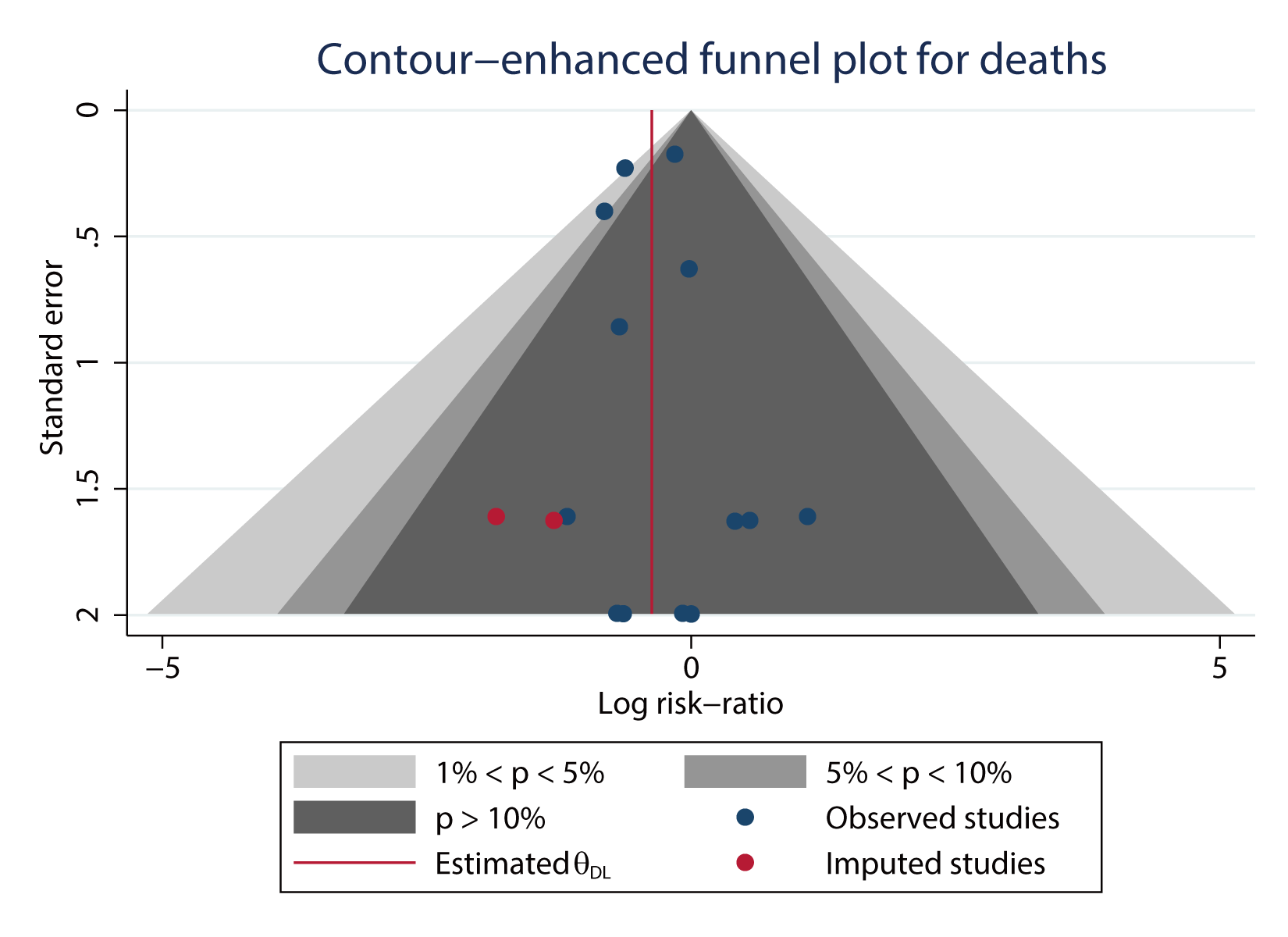

Supplement: S2 Fig — (TIF) [file pone.0262702.s003.tif]

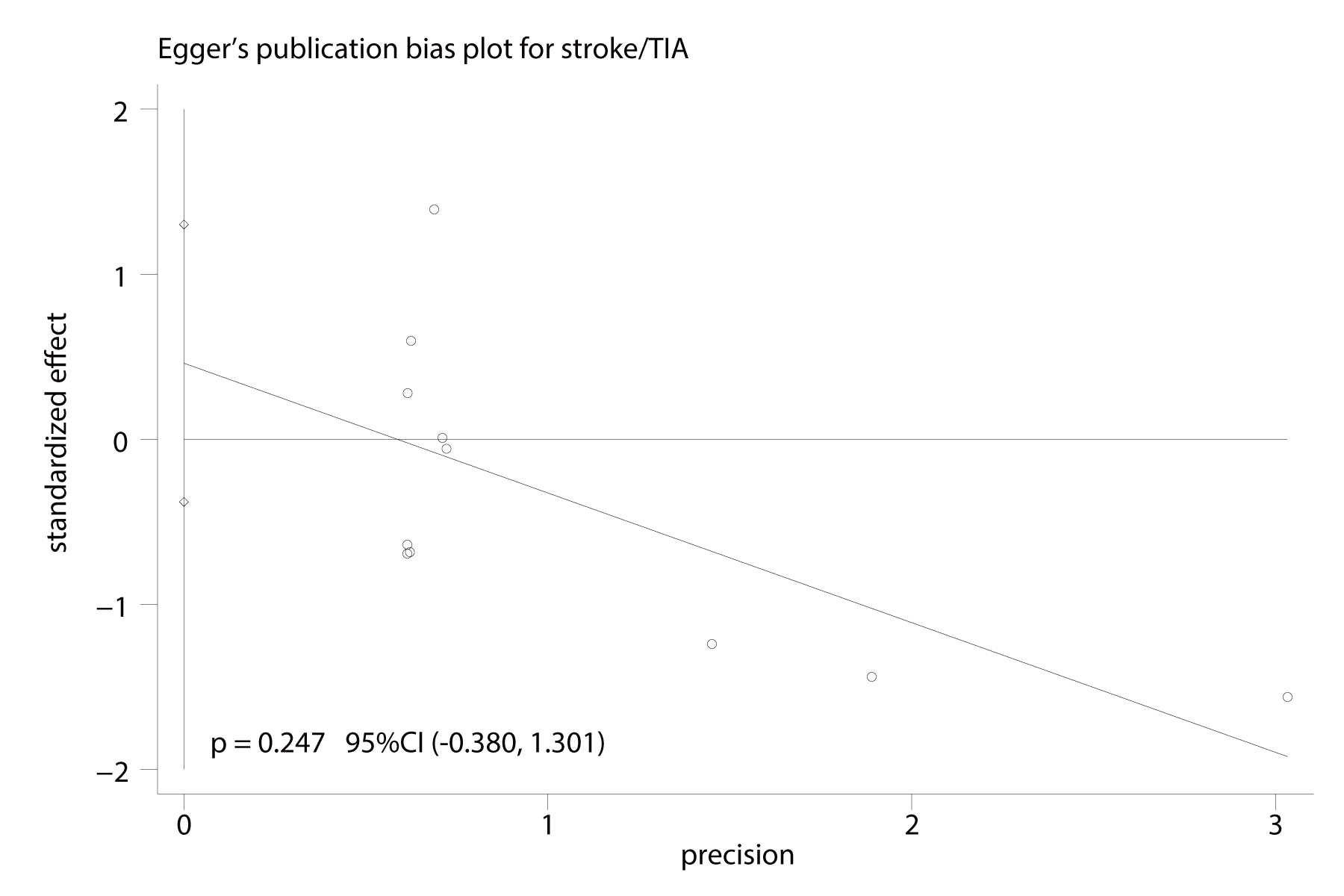

Supplement: S3 Fig — (TIF) [file pone.0262702.s004.tif]

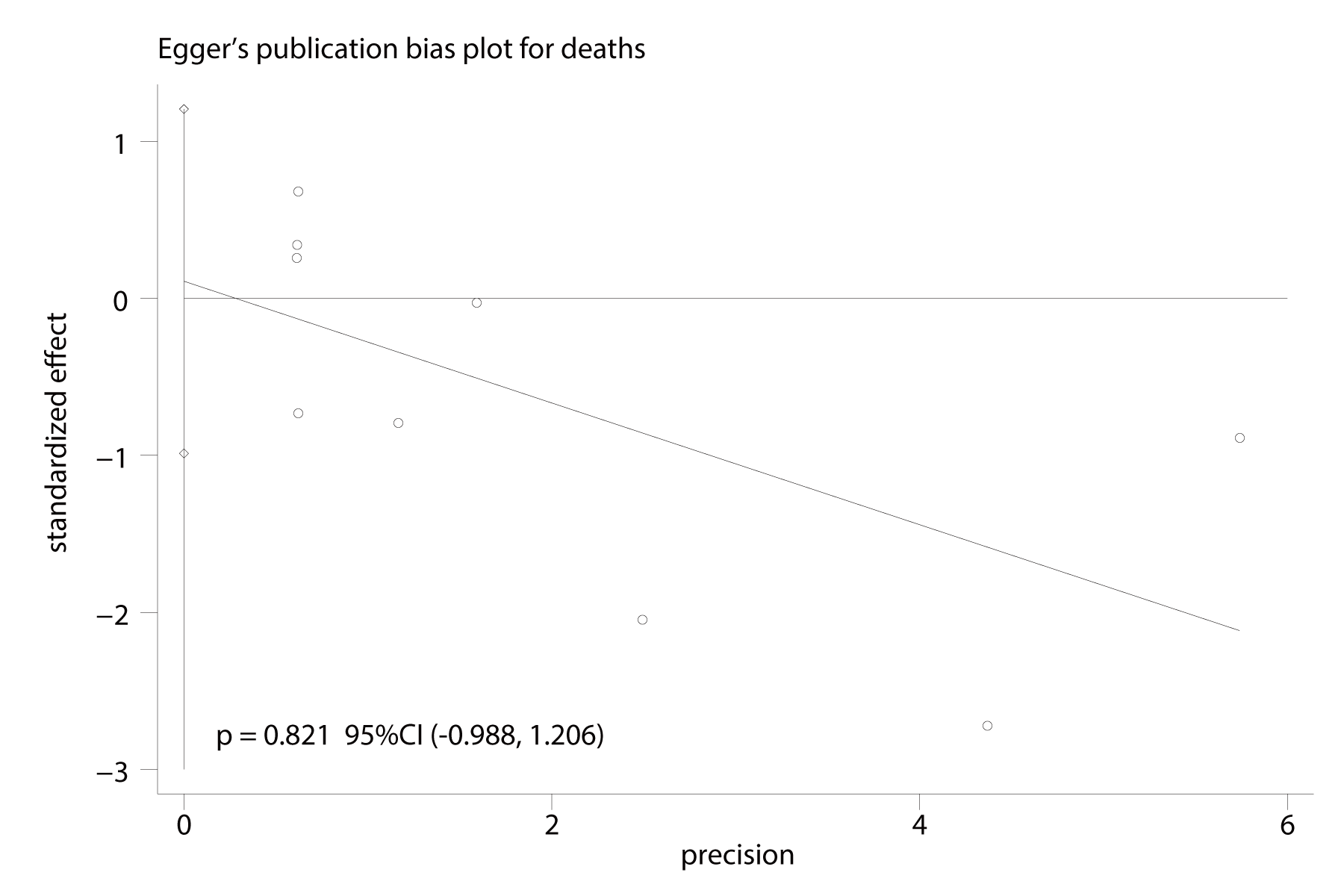

Supplement: S4 Fig — (TIF) [file pone.0262702.s005.tif]

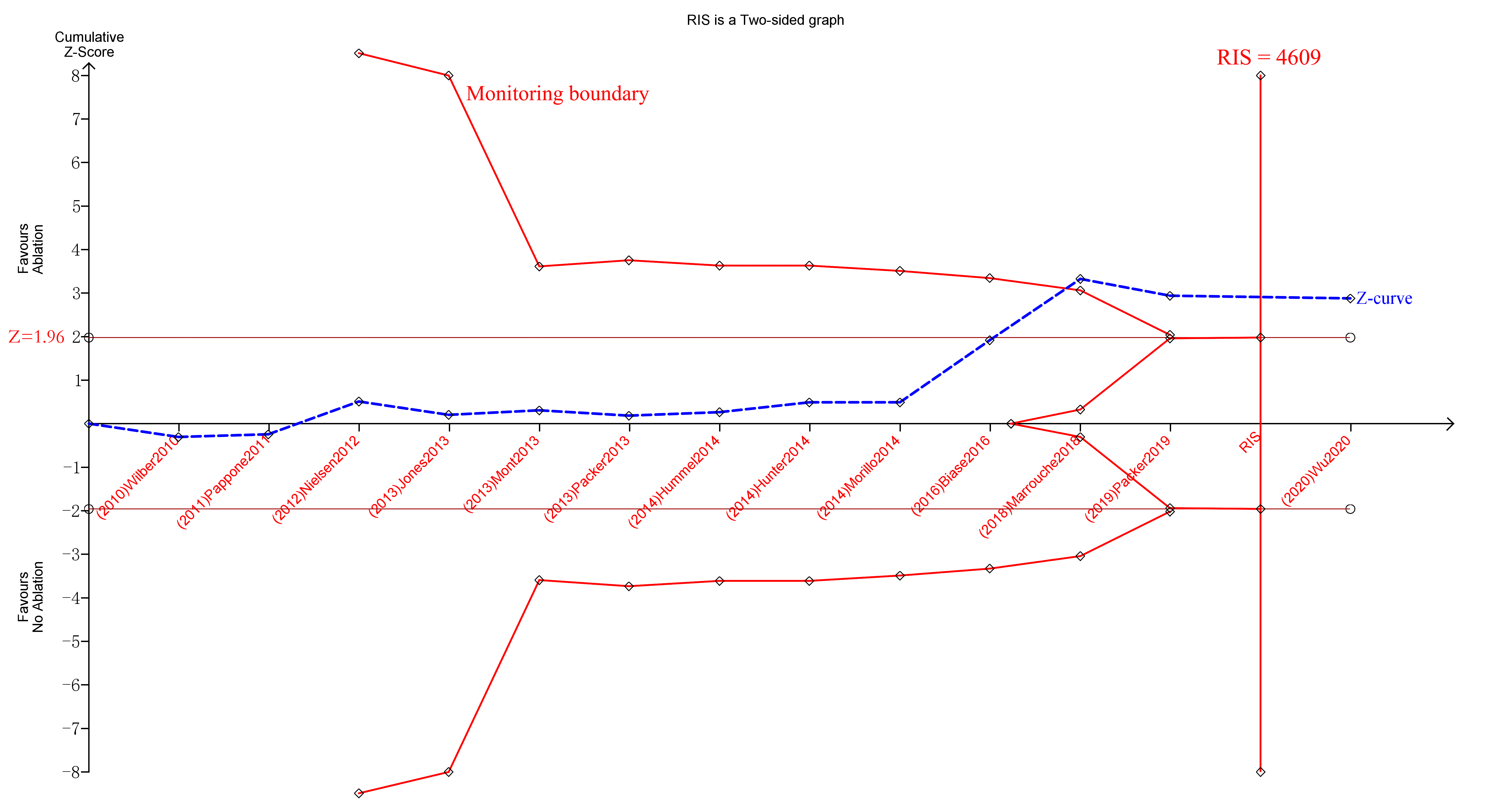

Supplement: S5 Fig — (TIF) [file pone.0262702.s006.tif]

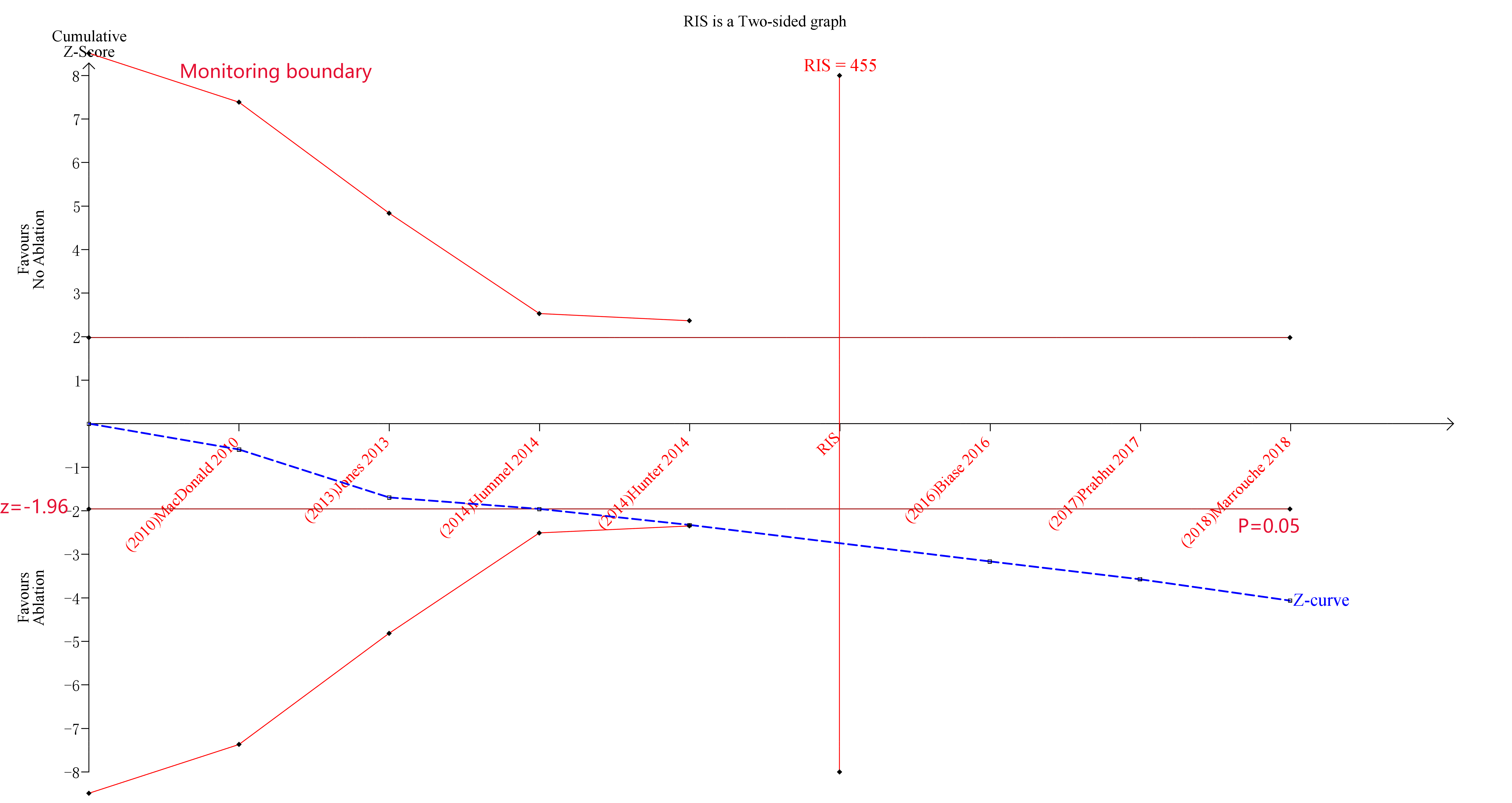

Supplement: S6 Fig — (TIF) [file pone.0262702.s007.tif]

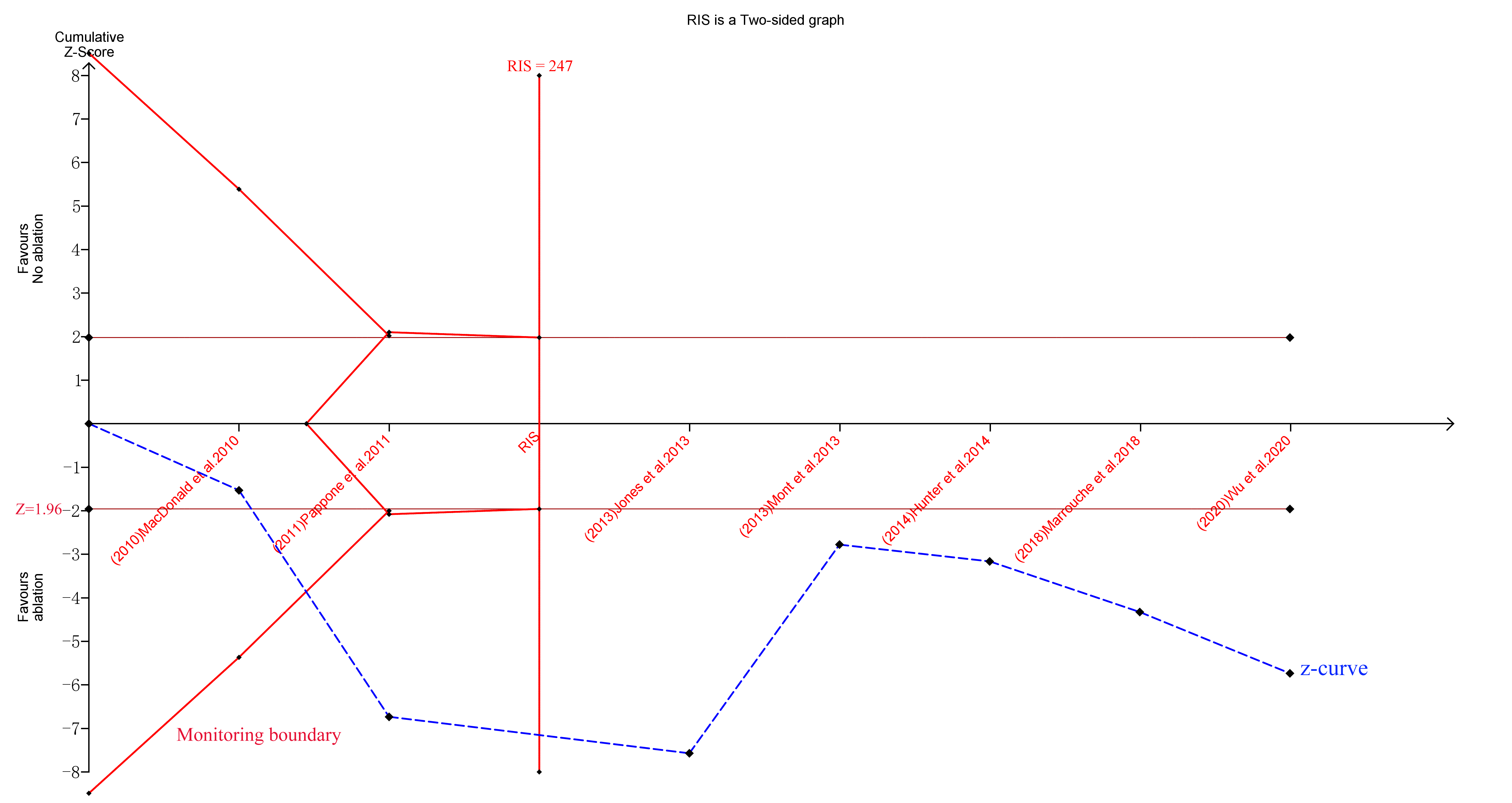

Supplement: S7 Fig — (TIF) [file pone.0262702.s008.tif]
