# Supplementary material for: The comparison of catheter ablation on hard outcomes versus medical treatment for atrial fibrillation patients: A meta-analysis of randomized, controlled trials with trial sequential analysis
Source: PLoS One. 2022 Jan 19;17(1):e0262702. doi: 10.1371/journal.pone.0262702 (PMC8769301; doi:10.1371/journal.pone.0262702)
Supplement: S1 Table — (DOCX) [file pone.0262702.s011.docx]

Supplementary Table S1. PubMed search strategy for trials comparing atrial fibrillation ablation with drug therapy

| Search  Number | Description | Number of Publications |
| --- | --- | --- |
| 1 | "atrial fibrillation"[MeSH Terms] | 57,945 |
| 2 | "atrial fibrillation*"[Title/Abstract] OR "auricular fibrillation*"[Title/Abstract] OR "atrium fibrillation*"[Title/Abstract] OR "af"[Title/Abstract] OR "a-fib"[Title/Abstract] | 92,701 |
| 3 | (#1 OR #2) | 102,906 |
| 4 | "catheter ablation"[MeSH Terms] | 33,297 |
| 5 | "catheter ablat*"[Title/Abstract] OR "catheter isolat*"[Title/Abstract] OR "transcatheter ablat*"[Title/Abstract] OR ("transcatheter"[All Fields] AND "isolat*"[Title/Abstract]) | 15,415 |
| 6 | (#4 OR #5) | 38,908 |
| 7 | "anti-arrhythmia agents"[MeSH Terms] | 27,725 |
| 8 | "anti-arrhythmia agents"[Title/Abstract] OR "antiarrhythmi*"[Title/Abstract] OR "anti arrhythmi*"[Title/Abstract] OR "procainamide"[Title/Abstract] OR "disopyramide"[Title/Abstract]OR"quinidine"[Title/Abstract] OR "mexiletine"[Title/Abstract] OR "flecainide"[Title/Abstract] OR "propafenone"[Title/Abstract] OR "bisoprolol"[Title/Abstract] OR "esmolol"[Title/Abstract] OR "amiodarone"[Title/Abstract] OR "dofetilide"[Title/Abstract] OR "sotalol"[Title/Abstract] OR "azimilide"[Title/Abstract] OR "ibutilide"[Title/Abstract] OR "cibenzoline"[Title/Abstract] OR "moricizine"[Title/Abstract] | 45,680 |
| 9 | (#7 OR #8) | 57,953 |
| 10 | (#3 AND #6 AND #9) | 2,647 |
| 11 | ("Randomized Controlled Trial" [Publication Type] OR "Controlled Clinical Trial" [Publication Type] OR "Clinical Trials as Topic"[Mesh:NoExp] OR randomized[Title/Abstract] OR placebo [Title/Abstract] OR randomly[Title/Abstract] OR trial[Title/Abstract]) NOT ("Animals"[Mesh] NOT "Humans"[Mesh]) | 1,434,682 |
| 12 | (#10 AND #11) | 402 |

Date of search: February 7st, 2021
