# Supplementary material for: The comparison of catheter ablation on hard outcomes versus medical treatment for atrial fibrillation patients: A meta-analysis of randomized, controlled trials with trial sequential analysis
Source: PLoS One. 2022 Jan 19;17(1):e0262702. doi: 10.1371/journal.pone.0262702 (PMC8769301; doi:10.1371/journal.pone.0262702)
Supplement: S2 Table — (DOCX) [file pone.0262702.s012.docx]

Supplementary Table S2. Cochrane Central Register of Controlled Trials search strategy for trials comparing atrial fibrillation ablation with drug therapy

| Search  Number | Description | Number of Publications |
| --- | --- | --- |
| 1 | MeSH descriptor: [Atrial Fibrillation] explode all trees | 4731 |
| 2 | (atrial fibrillation*):ti,ab,kw OR (auricular fibrillation*):ti,ab,kw OR (atrium fibrillation*):ti,ab,kw OR (af):ti,ab,kw OR (a-fib):ti,ab,kw | 14922 |
| 3 | #1 OR #2 | 14922 |
| 4 | MeSH descriptor: [Catheter Ablation] explode all trees | 1464 |
| 5 | (catheter ablat*):ti,ab,kw OR (catheter isolat*):ti,ab,kw OR (transcatheter ablat*):ti,ab,kw OR (transcatheter isolat*):ti,ab,kw | 3848 |
| 6 | #4 OR #5 | 3850 |
| 7 | MeSH descriptor: [Anti-Arrhythmia Agents] explode all trees | 1853 |
| 8 | (antiarrhythmi*):ti,ab,kw OR (anti-arrhythmi*):ti,ab,kw OR (procainamide):ti,ab,kw OR (disopyramide):ti,ab,kw OR (quinidine):ti,ab,kw | 4694 |
| 9 | (mexiletine):ti,ab,kw OR (flecainide):ti,ab,kw OR (propafenone):ti,ab,kw OR (bisoprolol):ti,ab,kw OR (esmolol):ti,ab,kw | 2808 |
| 10 | (amiodarone):ti,ab,kw OR (dofetilide):ti,ab,kw OR (sotalol):ti,ab,kw OR (azimilide):ti,ab,kw OR (ibutilide):ti,ab,kw | 2243 |
| 11 | (cibenzoline):ti,ab,kw OR (moricizine):ti,ab,kw | 116 |
| 12 | #7 OR #8 OR #9 OR #10 OR #11 | 7839 |
| 13 | #3 AND #6 AND #12 | 625 |

Date of search: February 7st, 2021
