# Supplementary material for: The comparison of catheter ablation on hard outcomes versus medical treatment for atrial fibrillation patients: A meta-analysis of randomized, controlled trials with trial sequential analysis
Source: PLoS One. 2022 Jan 19;17(1):e0262702. doi: 10.1371/journal.pone.0262702 (PMC8769301; doi:10.1371/journal.pone.0262702)
Supplement: S3 Table — (DOCX) [file pone.0262702.s013.docx]

Supplementary Table S3. Embase search strategy for trials comparing atrial fibrillation ablation with drug therapy

| Search  Number | Description | Number of Publications |
| --- | --- | --- |
| 1 | 'atrial fibrillation'/exp | 171,002 |
| 2 | 'atrial fibrillation*':ab,ti OR 'auricular fibrillation*':ab,ti OR 'atrium fibrillation*':ab,ti OR af:ab,ti OR a-fib:ab,ti | 161,126 |
| 3 | #1 OR #2 | 207,119 |
| 4 | 'catheter ablation'/exp | 35,939 |
| 5 | 'catheter ablat*':ab,ti OR 'catheter isolat*':ab,ti OR 'transcatheter ablat*':ab,ti OR 'transcatheter isolat*':ab,ti | 21,778 |
| 6 | #4 OR #5 | 38,273 |
| 7 | 'antiarrhythmic agent'/exp | 382,444 |
| 8 | antiarrhythmi*:ab,ti OR anti‐arrhythmi*:ab,ti OR procainamide:ab,ti OR disopyramide:ab,ti OR quinidine:ab,ti OR mexiletine:ab,ti OR flecainide:ab,ti OR propafenone:ab,ti OR bisoprolol:ab,ti OR esmolol:ab,ti OR amiodarone:ab,ti OR dofetilide:ab,ti OR sotalol:ab,ti OR azimilide:ab,ti OR ibutilide:ab,ti OR cibenzoline:ab,ti OR moricizine:ab,ti | 62,311 |
| 9 | #7 OR #8 | 392,876 |
| 10 | #3 AND #6 AND #9 | 5,317 |
| 11 | 'crossover procedure':de OR 'double-blind procedure':de OR 'randomized controlled trial':de OR 'single-blind procedure':de OR random*:de,ab,ti OR factorial*:de,ab,ti OR crossover*:de,ab,ti OR ((cross NEXT/1 over*):de,ab,ti) OR placebo*:de,ab,ti OR ((doubl* NEAR/1 blind*):de,ab,ti) OR ((singl* NEAR/1 blind*):de,ab,ti) OR assign*:de,ab,ti OR allocat*:de,ab,ti OR volunteer*:de,ab,ti | 2,733,512 |
| 12 | #10 AND #11 | 1,024 |
| 13 | #12 AND (2010:py OR 2011:py OR 2012:py OR 2013:py OR 2014:py OR 2015:py OR 2016:py OR 2017:py OR 2018:py OR 2019:py OR 2020:py) | 798 |

Date of search: February 7st, 2021
